# Transcriptome analysis uncovers key regulatory and metabolic aspects of soybean embryonic axes during germination

**DOI:** 10.1038/srep36009

**Published:** 2016-11-08

**Authors:** Daniel Bellieny-Rabelo, Eduardo Alves Gamosa de Oliveira, Elane da Silva Ribeiro, Evenilton Pessoa Costa, Antônia Elenir Amâncio Oliveira, Thiago Motta Venancio

**Affiliations:** 1 Laboratório de Química e Função de Proteínas e Peptídeos, Centro de Biociências e Biotecnologia, Universidade Estadual do Norte Fluminense Darcy Ribeiro, Campos dos Goytacazes, Brazil; 2 Núcleo em Ecologia e Desenvolvimento Sócio-Ambiental de Macaé (NUPEM), Campus UFRJ Macaé, Macaé, Brazil; 3 Unidade de Experimentação Animal, Universidade Estadual do Norte Fluminense Darcy Ribeiro, Campos dos Goytacazes, Brazil

## Abstract

Soybean (*Glycine max*) is a major legume crop worldwide, providing a critical source of protein and oil. The release of the soybean genome fuelled several transcriptome projects comprising multiple developmental stages and environmental conditions. Nevertheless, the global transcriptional patterns of embryonic axes during germination remain unknown. Here we report the analysis of ~1.58 billion RNA-Seq reads from soybean embryonic axes at five germination stages. Our results support the early activation of processes that are critical for germination, such as glycolysis, Krebs cycle and cell wall remodelling. Strikingly, only 3 hours after imbibition there is a preferential up-regulation of protein kinases and transcription factors, particularly from the LOB domain family, implying that transcriptional and post-transcriptional regulation play major roles early after imbibition. Lipid mobilization and glyoxylate pathways are also transcriptionally active in the embryonic axes, indicating that the local catabolism of oil reserves in the embryonic axes contributes to energy production during germination. We also present evidence supporting abscisic acid inactivation and the up-regulation of gibberellin, ethylene and brassinosteroid pathways. Further, there is a remarkable differential activation of paralogous genes in these hormone signalling pathways. Taken together, our results provide insights on the regulation and biochemistry of soybean germination.

Soybean [*Glycine max* (L.) Merr.] is the most economically important legume crop and a key factor of global food security due to its rich oil and protein content^1^. *G. max* belongs to the family Fabaceae, which is the third largest angiosperm family, comprising nearly 19,500 species^2^. Over the past 10 years, several legume species had their genomes sequenced, such as soybean^3^, common bean (*Phaseolus vulgaris*)^4^, chickpea (*Cicer arietinum*)^5^ and the diploid ancestors of the cultivated peanut^6^.

During maturation, cotyledonary cells accumulate massive amounts of reserves and other macromolecules that will be necessary during germination^7^. Conversely, the embryonic axis is not a primary source of reserves; instead, it harbours organs’ primordia, namely the shoot apex, hypocotyl and radicle^8^. Nevertheless, the embryonic axes of some species can grow and develop seedlings in the absence of cotyledons^9^, mainly because of stored maternal mRNAs, minor energetic reserves and enzymes that allow early metabolic resumption and growth upon imbibition^10^. During development, seed coat formation and progressive desiccation result in the cessation of metabolic activity. The period between the completion of seed development and germination is called after-ripening, a stage that results in increased sensitivity to factors that promote germination and decreased sensitivity to those that repress it^9^. When seeds are non-dormant and favourable environment conditions are met (e.g. when water and oxygen reach the embryo), germination starts and embryonic tissues resume growth^10^. Germination can be divided in early and late phases; the former comprises imbibition, DNA and organelle repair, metabolism reactivation, endosperm weakening and embryo elongation; whereas the latter involves radicle protrusion and rupture of the endosperm and seed coat^8^,^10^. Maintenance and release of seed dormancy are promoted by abscisic acid (ABA) and gibberellic acid (GA), respectively^8^,^10^,^11^; further, key genes involved in the GA/ABA interplay have been characterized [e.g. 9-*cis* epoxycarotenoid dioxygenase (*NCED*), gibberellin 2-oxidase (*GA2ox*), gibberellin insensitive dwarf 1 (*GID1*) and phytochrome-interacting factors (PIF’s)], which helped elucidate underlying aspects of ABA and GA regulation^12^,^13^,^14^.

Several large-scale transcriptomic studies were conducted for a variety of dicot species, such as *Arabidopsis thaliana*^15^ and chickpea^16^. Further, soybean transcriptomic studies allowed the discovery of important genes involved in the determination of floral organs and maintenance of shoot apical meristem^17^; seed filling^18^; response against drought stress^19^ and regulation of seed development^20^. Others found stage-specific alternative splicing isoforms of important genes^21^. However, the transcriptomic changes of soybean embryonic axes during germination remain unexplored, in spite of the essential nature of this stage in plant growth. Here we report the analysis of ~1.58 billion RNA-Seq reads from soybean embryonic axes in 5 seed germination time points. Among our main results are: i) a strong preferential activation of transcription factors (TFs) and protein kinases in the onset of germination; ii) an important carbohydrate partitioning system underlying cell wall metabolism and ATP production; iii) an early activation of primary catabolic pathways; iv) a strong up-regulation of genes involved in hormone biosynthesis, catabolism and signalling; iv) transcriptional divergence and potential sub-functionalization of paralogous genes with critical roles in hormone signalling, (e.g. *GID1, DELLA* and *CYP707A*).

## Results and Discussion

### Transcriptome sequencing of embryonic axes during soybean germination

Aiming to understand the transcriptional landscape of soybean embryonic axes during germination, we sequenced mRNA libraries of embryonic axes at 5 different germination time points: dry (0 hours after imbibition, HAI), 3-HAI, 6-HAI, 12-HAI and 24-HAI. A total of 242 to 372 million paired-end reads were sequenced per stage (Table 1). In terms of depth, our dataset (~1,58 billion reads) is ~2.4 times greater than that produced by a recent *G. max* RNA-Seq study^21^. The data reported here are also of good quality, as 91.8% (~1.45 billion) of the reads were successfully mapped on the soybean reference genome, out of which ~1,03 billion (~71.0%) were uniquely mapped (Table 1). We found detectable transcription (FPKM > 1; see methods for details) for 33,305 (61.48%) genes predicted in the *G. max* reference genome (Table S1).

**Figure 1:**
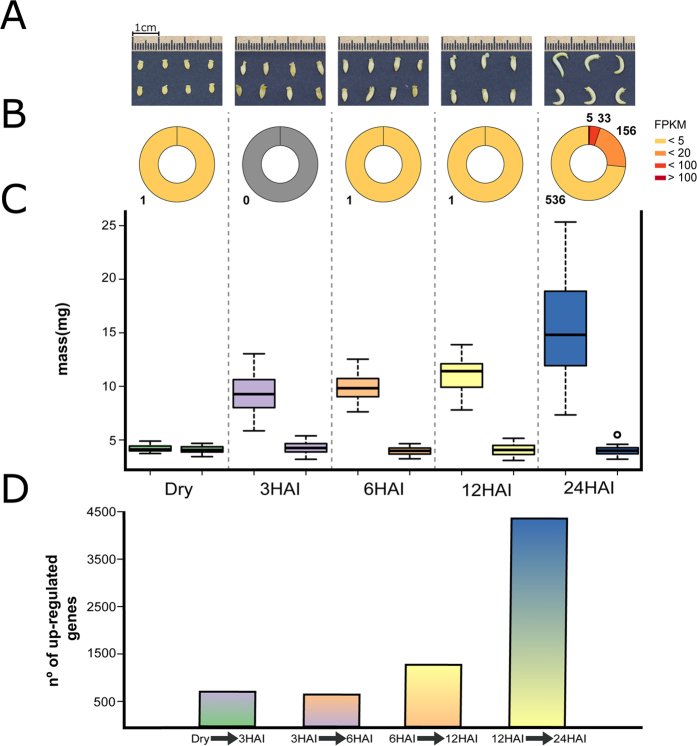
(**A**) Photographs of soybean embryonic axes at 0- (dry), 3-, 6-, 12- and 24-HAI; (**B**) Number of preferentially expresses genes and their transcriptional levels (in FPKM); (**C**) Distribution of fresh and dry weight of embryonic axes (20 seeds per condition); (**D**) Number of up-regulated genes between sequential germination time points.

Our results support the notion that the mRNA populations of early germinating seeds comprise two distinct subpopulations: transcripts that are important at late maturation (e.g. Late Embryogenesis Abundant proteins, LEAs) and; transcripts that will be required for early germination phases (Table S1; Figures S1 and S2)^8^,^10^,^22^. In an initial analysis, we found that 99.6% of the preferentially expressed genes (730 out of 733; see methods for details) are remarkably activated at 24-HAI (Fig. 1A; Table S2). Although it would be intuitive to associate this dramatic shift with major morphological transitions, no significant dry weight changes were observed (Fig. 1), implying that the embryonic axis growth during germination is mainly driven by water intake and cell expansion^8^. Photosynthesis (GO:0015979) and response to oxidative stress (GO:0006979) genes are remarkably present in this 24-HAI preferentially expressed gene set. We hypothesize that this ~700-fold enrichment in preferentially expressed genes constitutes a preparation phase for seedling establishment and autotrophic seedling growth (discussed later).

### Genes involved in cell-wall homeostasis, protein kinases and TFs are up-regulated early during germination

We detected 730, 674, 1307 and 4394 up-regulated genes in the dry to 3-HAI, 3-HAI to 6-HAI, 6-HAI to 12-HAI and 12-HAI to 24-HAI intervals, respectively. To uncover major trends in these gene sets, we performed a Gene Ontology (GO) enrichment analysis using stringent statistical criteria (Table S3). This analysis revealed an early (i.e. at 3-HAI) significant up-regulation of cell wall genes (GO:0005618; 14 genes), particularly xyloglucan endotransglucosylases (GO:0016762; 10 genes; e.g. *Glyma11g36730*, 77-fold up-regulated), which promote cellular expansion by remodelling cellulose microfibrils. Many other cell wall genes were up-regulated at 24-HAI (43 genes) (Table S3), although this second gene set includes 15 cellulose synthases, which are critical for cell wall homeostasis. There was also a clear enrichment of genes annotated with the general term GO:0005975 (i.e. “carbohydrate metabolic process”) at 24-HAI (Table S3), encompassing several gene families involved in cell wall organization or defence mechanisms (e.g. beta-glucosidases and pectin lyases).

The only GO term significantly enriched at all stages was GO:0016705, which comprises a variety of oxidoreductase families, such as: 1) ACC oxidases ACO1 and ACO4, which catalyse the last step in ethylene biosynthesis. Interestingly, 5 out of 6 differentially expressed ACO4 genes reach high expression levels (FPKM > 10) only after 6-HAI, indicating that ethylene is more relevant later in germination^23^; 2) 47 cytochrome P450 genes (i.e. CYP), which are mainly induced between 12 and 24-HAI (33 genes); 3) GA oxidases (8 genes) and; 4) 28 oxoglutarate/iron-dependent dioxygenases. Further, we found a related GO term, GO:0055114 (oxidation-reduction process), enriched among genes induced at 6-, 12- and 24-HAI; this group includes aldehyde dehydrogenases, FAD-binding Berberine family proteins, lipoxygenases, peroxidases, Sks cell wall oxidoreductases and rhamnose synthases. Although structurally distinct, several of these families have been linked with cell wall metabolism and homeostasis^24^,^25^,^26^.

We also found a remarkable enrichment of several GO terms related to photosynthesis (e.g. GO:0009579, thylakoid; GO:0015995, chlorophyll biosynthetic process), which we unified under the GO term “photosynthesis” (GO:0015979). This large group (77 genes induced at 12- and/or 24-HAI) comprise multiple photosystem I and II subunits, including genes encoding proteins of the light-harvesting complex and chlorophyll binding proteins. Many of these genes are also preferentially expressed at 24-HAI (discussed above) and their expression late in germination probably integrate a preparation phase for the transition to autotrophic growth.

The GO enrichment analyses also pointed us to two remarkable aspects of the soybean germination transcriptome: the preferential up-regulation of genes encoding protein kinases (GO:0004672) and transcriptional regulators (GO:0030528) at 3-HAI (Table S3). Although most of the up-regulated protein kinases are transcribed at moderate levels (42 genes; FPKM < 10), 7 were highly transcribed (FPKM > 10): *Glyma09g32390* (a proline extensin-like receptor kinase) and *Glyma07g38510* (a mitogen-activated kinase) are not well characterized and their early up-regulation is followed by a subsequent down-regulation, suggesting specific roles in the germination onset. *Glyma06g15270* is a brassinosteroid (BR) receptor (homolog of ATBRI1/AT4G39400) that is 4-fold up-regulated at 3-HAI. BR signalling via ATBRI1 has been shown to promote germination by countering ABA effects^27^. Further, *Glyma06g15270* is up-regulated in response to exogenous BR and is able to restore the *Arabidopsis* dwarf mutant bri1-5 phenotype^28^. Strikingly, *Glyma12g22660*, the homolog of the *Arabidopsis THESEUS1*, is also up-regulated at 3-HAI. *THESEUS1* is induced by BR and is required for optimal cell elongation^29^. Further, *THESEUS1* attenuates the inhibition of hypocotyl growth in some cell-wall mutants^30^ and integrates a cell-wall surveillance system in expanding cells^30^. Interestingly, *Glyma12g22660* is the only one of these 7 highly up-regulated protein kinases that is also significantly induced between 12 and 24-HAI. This bimodal pattern of activation at 3- and 24-HAI coincides with the second water uptake phase and with a massive up-regulation of cell wall genes (Table S3). Therefore, we hypothesize that BR signalling via *BRI1* and *THESEUS1* constitutes an important system that regulates cell expansion during germination. Another kinase that is highly induced at 3-HAI is *Glyma10g33240*, an ethylene receptor whose homolog functions in *Arabidopsis* seedlings^31^. The sixth up-regulated kinase is *Glyma14g39290*, a homolog of the *Arabidopsis* transmembrane kinase *TMK1 (AT1G66150*), reported to be transcriptionally induced by GA^32^. Finally, we found an apparently unexpected up-regulation of *Glyma07g10695*, a homolog of the *Arabidopsis AT1G18390,* which is positively involved in ABA signalling^33^. Overall, our results suggest that signalling cascades involving protein phosphorylation play critical roles in the germination onset.

Perhaps the most remarkable aspect of the genes up-regulated at 3-HAI is the massive presence of DNA-dependent transcriptional regulators (GO:0006355) (Table S3), most of which annotated as DNA-binding TFs in PlantTFDB^34^. Since PlantTFDB is a public repository specialized on TFs, we analysed the differential expression of TFs as per this database classifications. We detected transcription of 53.8% of the soybean TFs (2000/3717) during germination, out of which 432 were significantly up-regulated in at least one stage. Notably, 3-HAI was the only stage with significant over-representation of TFs (81 genes; p-value = 8 × 10^−8^; Fisher Exact Test), corroborating the GO enrichment results. Overall, ~11% of the genes up-regulated at 3-HAI are TFs, which allowed us to uncover other remarkable trends: 1) the striking over-representation of LOB domain genes induced early during germination^35^; 9 out of 81 TFs up-regulated at 3-HAI are from the LOB family, which has a total of 36 members expressed in at least one germination time point. The transcriptional fold-changes of the LOB genes are also very high; for example, *Glyma07g10180* and *Glyma13g07590* have low expression levels (<2 FPKM) in dry seeds and are greatly up-regulated by 3-HAI (>96 FPKM). These observations are coherent with our previous discussion on BR signalling, as BRs induce the expression of LOB TFs, which in turn repress the accumulation of BRs via up-regulation of the BR-inactivating enzyme BAS1^36^. Therefore, LOB TFs are part of a feedback loop involved in BR homeostasis^37^. Nevertheless, because *BAS1* genes were transcribed at low levels during soybean germination, this feedback loop is either inactive or involves other genes at this phase. Further, LOB TFs promote the transcription of an expansin and probably other cell wall-loosening genes^38^. Thus, we hypothesize that LOB TFs regulate cell-wall homeostasis during germination; 2) the 3.2-fold up-regulation of *Glyma02g08835*, an ethylene response factor gene. *Glyma02g08835* is highly expressed in dry seeds (FPKM = 72.9), further up-regulated at 3-HAI (FPKM = 237) and sharply down-regulated at 24-HAI (FPKM = 7.55), suggesting that it is specifically required in the onset of germination. Interestingly, this transient up-regulation seems to be a general trend of ERF TFs; 9 out of the 13 ERFs that are up-regulated at 3-HAI have their levels reduced by at least 2-fold by 24-HAI; 3) Finally, we have also noticed the complete absence of up-regulated TFs from several large families (e.g. AP2, TALE, FAR1, G2-like, C3H and MYB_related) at 3-HAI, which uncovered a clearly biased activation of particular families at this stage: 69% (56/81) of the TFs up-regulated at 3-HAI belong to only 12.7% of the TF families that have at least one member transcribed during germination (ERF: 13, LOB: 9, HD-ZIP: 9, bZIP: 7, bHLH: 7, GRAS: 6, Dof: 5).

In addition to TFs, chromatin proteins are also extremely relevant in regulating gene expression on a large scale. We found enrichment for the term GO:0000785 (i.e. chromatin) (Table S3) among genes up-regulated at 24-HAI; this group comprises 41 genes, out of which 36 encode histone subunits and 5 encode high mobility group A genes (HMGA), which are key non-histone proteins that contain AT-hook motifs^39^,^40^. The up-regulation of these genes late in germination may be important for the chromatin structure changes underlying DNA duplication and cell division that take place during early seedling growth.

### Transcriptional activation of primary metabolism genes during germination

**Figure 2:**
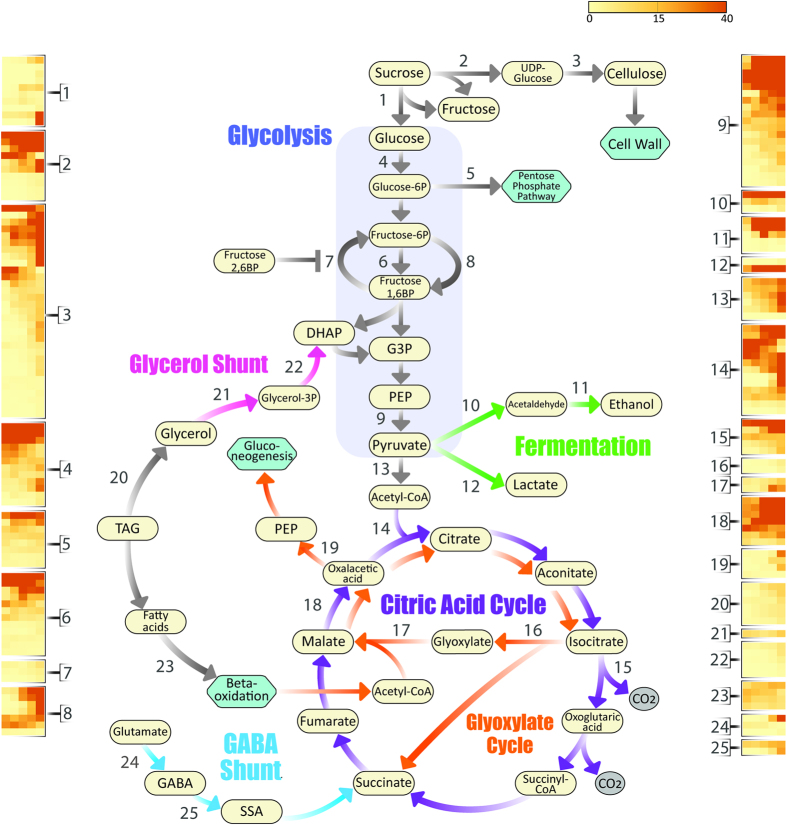
Major metabolic pathways and enzymes discussed in this study. Transcriptional levels of key genes are represented in heatmaps. Genes are numbered as following: 1) invertase; 2) sucrose synthase (SuSy); 3) cellulose synthase; 4) hexokinase (HK); 5) glucose-6-phosphate dehydrogenase (G6PDH); 6) phosphofructokinase (PFK); 7) Fructose 1,6-bisphosphatase (FBPase); 8) diphosphate-fructose-6-phosphate 1-phosphotransferase (PFP); 9) pyruvate kinase (PK); 10) pyruvate decarboxylase; 11) alcohol dehydrogenase (ADH); 12) lactate dehydrogenase (LDH); 13) pyruvate dehydrogenase subunit E2 (PDH-E2); 14) citrate synthase (CSY); 15) isocitrate dehydrogenase (IDH); 16) isocitrate lyase (ICL); 17) malate synthase (MSY); 18) malate dehydrogenase (MDH); 19) phosphoenolpyruvate carboxykinase (PEPCK); 20) triacylglycerol (TAG) lipase; 21) glycerol kinase; 22) glycerol-3-phosphate dehydrogenase (GPDHC1); 23) COMATOSE (CTS); 24) glutamate decarboxylase (GAD); 25) gamma-amino-N-butyrate transaminase (GABA-T). The 5 columns in each heatmap represent the germination time points (dry; 3-HAI; 6-HAI; 12-HAI; 24-HAI). Other abbreviations: G3P: glucose-3-phosphate; DHAP: dihydroxyacetone phosphate; PEP: phosphoenolpyruvate; SSA: succinic semialdehyde.

Although most of the stored reserves are mobilized after seed germination, catabolic pathways must be activated early upon imbibition to sustain axis growth. However, the relative contribution and transcriptional regulation of each pathway to the metabolism of the embryonic axis are not fully understood. Sucrose and raffinose family oligosaccharides (RFOs) are the main soluble carbohydrates available in soybean seeds^41^ and are mainly accumulated during desiccation^42^. RFO concentrations sharply decline during germination, concomitantly with a loss of desiccation tolerance and an increase in the levels of reducing sugars^43^. In terms of energy production, invertases are the major drivers of sucrose usage. We found 10 expressed invertases, out of which 6 were induced at 24-HAI and 4 showed high expression levels at this stage (Fig. 2; Table S4). These results support an increasing demand for sucrose-derived monosaccharides later in germination and probably during early seedling growth.

Sucrose can also be cleaved in a reversible reaction catalysed by sucrose synthase (SuSy; Fig. 2). While invertases are associated with high energy demand conditions (e.g. cell expansion), SuSy activity has been linked with the biosynthesis of structural or reserve polysaccharides^44^. Although reversible, SuSy activity favours sucrose cleavage in most heterotrophic tissues, providing sugar-nucleotide precursors for structural and storage polysaccharide biosynthesis^44^. Further, SuSy can associate with membranes^44^ and channel UDP-glucose to cellulose synthase complexes^45^, which interact with microtubules and guide cellulose deposition and cellular elongation^46^. Six and 19 SuSy and cellulose synthase genes were highly expressed at 24-HAI, respectively (Fig. 2; Table S4). As discussed above, 14 of these 19 cellulose synthase genes were up-regulated at 24-HAI (Table S3). The complex transcriptional patterns of invertase and SuSy genes probably constitute an intricate system of sucrose partitioning between ATP production from reducing sugars and cellulose biosynthesis from UDP-glucose, which are two essential processes underlying cellular expansion during germination. Finally, in spite of the intense sucrose degradation supported by our results, it has been long shown that sucrose concentrations do not decrease during germination^41^,^43^, suggesting a renewal of the embryonic axes’ sucrose pools, probably from the degradation of other reserves (e.g. oil).

We also found an early activation of glycolysis and respiratory pathways, which could be partially fed by sucrose degradation. Among these genes are: 12 hexokinases; 12 phosphofructokinases (PFK); 13 glyceraldehyde 3-phosphate dehydrogenases (GAPDH); triosephosphate isomerases (TIM) and pyruvate kinases (PKs) (Fig. 2; Table S4). The *A. thaliana At2g21170* encodes a plastid TIM that is required for heterotrophic to autotrophic growth in post-germinative stages^47^. We found two highly expressed *At2g21170* orthologs during soybean germination (Fig. 2), suggesting that this gene could also be critical in the transition to autotrophic growth in soybean. Glucose can also be consumed by the pentose phosphate pathway, which has a committed step catalysed by glucose-6-phosphate dehydrogenase (G6PDH) (Fig. 2). Eight G6PDH genes were transcribed during germination (Fig. 2), indicating that glucose is partially channelled to the pentose phosphate pathway during soybean germination. Further, we found moderate transcription levels of 3 fructose-1,6-bisphosphatases (FBPase), which catalyse the reverse reaction of PFK, converting fructose 1,6-bisphosphate to fructose-6-phosphate in the Calvin Cycle and gluconeogenesis (Fig. 2; Table S4). This interconversion is tightly regulated by fructose 2,6-bisphosphate (Fru2,6P_2_), which activates PFK and represses FBPase in non-plant organisms^48^. In plants, however, Fru2,6P_2_ activates a pyrophosphate dependent PFK (PFP), whereas the ATP-dependent PFK is largely insensitive to Fru2,6P_2_^48^. Seven PFP genes are expressed during germination, suggesting a complex interplay between PFKs and PFPs (Fig. 2; Table S4). Overall, our results support an intense carbohydrate catabolism via glycolysis/TCA cycle and pentose phosphate pathway. Nevertheless, the expression of FBPase and two critical sucrose biosynthesis enzymes [i.e. sucrose-phosphate synthase (e.g. *Glyma13g23060*) and sucrose-phosphate phosphatase (e.g. *Glyma20g16990*)] should not be neglected, as fluctuations in Fru2,6P_2_ concentrations may trigger sucrose biosynthesis in particular conditions/sites. The expression of sucrose biosynthesis enzymes may contribute to the regeneration of the sucrose pools discussed above.

Acetyl-CoA is central for energy metabolism, particularly in: 1) mitochondria, where it is oxidized via the TCA cycle and; 2) glyoxysomes, where it is an intermediate in the conversion of fatty acids to carbohydrates. Acetyl-CoA can be produced through a transacylation reaction catalysed by the pyruvate dehydrogenase E2 subunit of the pyruvate dehydrogenase complex (PDC). We found 5 PDC-E2 genes for which transcription increases as germination proceeds, suggesting that aerobic metabolism resumes very early in germination (Fig. 2; Table S4). Further, PDH-E1s (19) and PDH-E3s (7) PDC subunits were also highly transcribed, as well as key TCA enzymes, such as citrate synthase (CSY; 13 genes), isocitrate dehydrogenase (IDH; 6 genes) and malate dehydrogenase (MDH; 8 genes) (Fig. 2; Table S4). Importantly, most of these TCA genes reach their maximum transcriptional levels at 24-HAI, which could explain the increased activity of respiratory pathways after mitochondrial repair and biogenesis, which take place early in germination^10^,^49^.

Despite the rapid water uptake and activation of genes involved in respiratory pathways, oxygen levels are often sub-optimal inside the seeds. Together with the presence of immature or damaged mitochondria in imbibed seeds, fermentation has been suggested to play central roles in the onset of germination^49^. In support of this hypothesis, we found highly expressed pyruvate decarboxylase, alcohol dehydrogenase (ADH) and lactate dehydrogenase (LDH) genes during germination (Fig. 2; Table S4). Strikingly, all these genes reach maximum transcriptional levels at 6-HAI, implying that fermentation is indeed more relevant before mitochondria are fully repaired or synthesized *de novo*.

Triacylglycerols stored in oil bodies are typically located close to glyoxysomes, organelles specialized in converting fatty acids derived from TAGs into soluble-sugar precursors via the glyoxylate cycle^50^. Although the glyoxylate and TCA cycles share several enzymes, the former bypasses the decarboxylation steps of the latter by the activity of isocitrate lyase (ICL) and malate synthase (MSY). Malate is then oxidized to oxaloacetate by malate dehydrogenase (MDH), which is subsequently converted to phosphoenolpyruvate (PEP) by phosphoenolpyruvate carboxykinase (PEPCK)^50^ (Fig. 2; Table S4). Interestingly, we found moderate expression levels for two ICL genes and high expression levels for two MSY genes, particularly the glyoxysomal MSY (*Glyma17g13730*) (Fig. 2; Table S4). Three MDH and 3 PEPCK genes reach their maximum expression levels at 24-HAI (Fig. 2; Table S4). Further, at least 6 TAG (GDSL-like) lipase and 4 COMATOSE genes^51^ were also expressed during germination, supporting the transport of fatty acyl-CoA into peroxisomes/glyoxysomes (Fig. 2; Table S4). The mobilization of triacylglycerols also generates significant amounts of glycerol, which can enter glycolysis as dihydroxyacetone phosphate (DHAP) via a glycerol shunt pathway involving glycerol kinase (GLI1) and glycerol-3-phosphate dehydrogenase (G3PDc)^10^. Accordingly, we found a GLI1 and 5 GPDHc genes transcribed during germination (Fig. 2; Table S4). It is widely accepted that the mobilization of oil reserves is more critical after germination^50^,^52^, although several studies demonstrated that peroxisomal β-oxidation and glyoxylate cycle determine germination potential under several conditions^53^,^54^. Further, the early activation of genes involved in lipid mobilization reported here is in accordance with a previous report that storage oil bodies localized at embryonic cells are locally catabolized during germination^49^.

Seed storage proteins are critical to provide amino acids for protein synthesis and energy production, particularly glutamate and aspartate, which are the most abundant amino acids in soybean seeds (FAO; http://www.fao.org/docrep/t0532e/t0532e02.htm). They are substrates of alanine and aspartate aminotransferases (AlaAT and AspAT), enzymes that are activated during imbibition and likely to play important roles in respiratory pathways^10^. Several lines of evidence support the importance of Asp accumulation in germination, such as the negative influence of ABA on AspAT protein levels and fast increase in AspAT transcription after imbibition^10^,^49^. Our results support the high activity of these enzymes, as 7 AspATs and 4 AlaATs are expressed throughout germination. The non-protein amino acid γ-aminobutyrate (GABA) also has important energetic roles during seed germination^10^,^49^, as demonstrated in *Arabidopsis*^52^ and chestnut^55^. Further, GABA accumulation in germinating soybean seeds is correlated with glutamate decarboxylase (GAD) activity^56^. GAD synthesizes GABA from glutamate and is the first enzyme in the GABA shunt pathway. GABA is then metabolized by GABA transaminases (GABA-T), generating succinic semialdehyde that enters the TCA as succinate (Fig. 2; Table S4). We found expressed GAD and GABA-T genes, supporting the activity of a GABA shunt during soybean germination (Fig. 2; Table S4).

It has been shown for several species (e.g. barley) that amino acids used for *de novo* protein synthesis come from the degradation of storage proteins^49^. However, we found strong transcriptional evidence supporting the activation of some amino acid biosynthesis pathways during germination. A crucial step in the biosynthesis of aromatic amino acids (i.e. phenylalanine, tyrosine and tryptophan) is the production of chorismate via the shikimate pathway, which starts with a rate-limiting reaction catalysed by 3-deoxy-7-phosphoheptulonate (DAHP) synthase: the conversion of PEP and erythrose 4-phosphate (from the pentose phosphate pathway) into 3-deoxy-D-arabino-hept-2-ulosonate 7-phosphate^57^. DAHP synthase links the carbohydrate metabolism to the shikimate pathway, which will ultimately produce chorismate, a central metabolite in the biosynthesis of aromatic amino acids, salicylate and B9 vitamin (folate)^57^. We found high transcriptional levels of a DAHP synthase throughout germination (*Glyma15g06020*, FPKM > 37), supporting that some carbon from the pentose phosphate pathway feed into the chorismate pathway. Other shikimate pathway genes were also highly expressed, such as shikimate kinase (e.g. *Glyma04g39700*) and chorismate synthase (*Glyma20g31980*). Since chorismate is a substrate for the synthesis of different metabolites, we investigated its possible routes in more detail. Firstly, we found 7 expressed chorismate mutase genes (e.g. *Glyma01g06660*), which catalyse the synthesis of prephenate, a substrate for Tyr and Phe biosynthesis. We have also found expression of arogenate dehydrogenases (e.g. *Glyma11g35760*) and arogenate dehydratases (e.g. *Glyma11g15750* and *Glyma17g05290*), which are required to produce Tyr and Phe, respectively. Further, we found two expressed aminodeoxychorismate synthases (i.e. *Glyma10g35580* and *Glyma20g31966*) and one 4-amino-4-deoxychorismate lyase (*Glyma12g35520*), indicating that folate biosynthesis from chorismate is also active during germination. Conversely, no isochorismate synthase gene is expressed, supporting the absence of salycylate biosynthesis from chorismate during germination. Although nuclear-encoded, the shikimate pathway and other biosynthetic processes take place at the plastids, which are largely non-functional in dry seeds. However, a recent study demonstrated that plastid biogenesis starts early in phase I of soybean germination^58^. Therefore, we hypothesize that newly generated plastids get promptly equipped with the shikimate and other biosynthetic pathways.

### Hormone biosynthesis and signalling during germination

The embryonic axis gives rise to the organ primordia and has primary regulatory roles in plant growth. In this section we explore the expression of genes involved in the biosynthesis and signalling of key hormones during germination. Seed desiccation and maintenance of dormancy are positively regulated by ABA^8^,^10^. ABA Insensitive3/Viviparous1 (ABI3/VP1) is a TF that mediates ABA responses by promoting storage reserve deposition, desiccation tolerance and seed dormancy^59^. We found two highly expressed ABI3 genes during germination (*Glyma08g47240* and *Glyma18g38490*, FPKM > 62; Table S1). In *Arabidopsis* and tomato, ABI3 expression is repressed soon after germination by an endospermic GA or GA-derived signal^60^. This also seems to be the case in soybean, as *Glyma08g47240* and *Glyma18g38490* remain highly expressed by 12-HAI, being 9 to 10-fold down-regulated by 24-HAI. ABI3 degradation is promoted by the AIP2 E3 ligase^61^, for which we found two expressed homologs (*Glyma17g33631* and *Glyma14g12380*). Interestingly, ABI5, a TF that acts downstream of ABI3^62^, is also highly expressed early during germination and repressed by 24-HAI. Similarly to ABI3, ABI5 is also down-regulated by proteolysis^63^. These results indicate that ubiquitin-mediated proteolysis of critical TFs involved in ABA response precedes their transcriptional repression, probably allowing a fine-tuned regulation of ABA effects.

ABA catabolism plays important roles in regulating germination potential, particularly by ABA 8′-hydroxylation catalysed by the CYP707A1-4 family^59^. CYP707A1 reduces ABA concentrations during mid-maturation, whereas CYP707A2 is more related to late-maturation and imbibition stages^64^. Accordingly, we found a CYP707A2 gene that is highly expressed in dry seeds and at 3-HAI (*Glyma09g41960*; FPKM > 91), being strongly down-regulated at 24-HAI (FPKM = 2.58). Other CYP707A genes display different expression patterns. Conversely, the CYP707A1 gene *Glyma16g20490* is transcribed below the detection threshold until 6-HAI, being up-regulated at 12 and 24-HAI. Given that *Glyma16g20490* is not expressed in dry seeds, it is likely to be transcriptionally inactive in late-maturation. Therefore, our results reveal the opposed and cyclic transcription of CYP707A1 and CYP707A2 genes during soybean maturation and germination, which could constitute a system to balance active ABA levels. Surprisingly, several other highly expressed ABA-signalling genes were also expressed during soybean germination (e.g. PYL genes: *Glyma16g02910*; HAI3 ABA-induced protein phosphatases: *Glyma01g43460*). Therefore, we hypothesize that post-translational regulation and ABA catabolism are the major mechanisms modulating ABA effects before germination-promoting transcriptional programs are fully installed.

**Figure 3:**
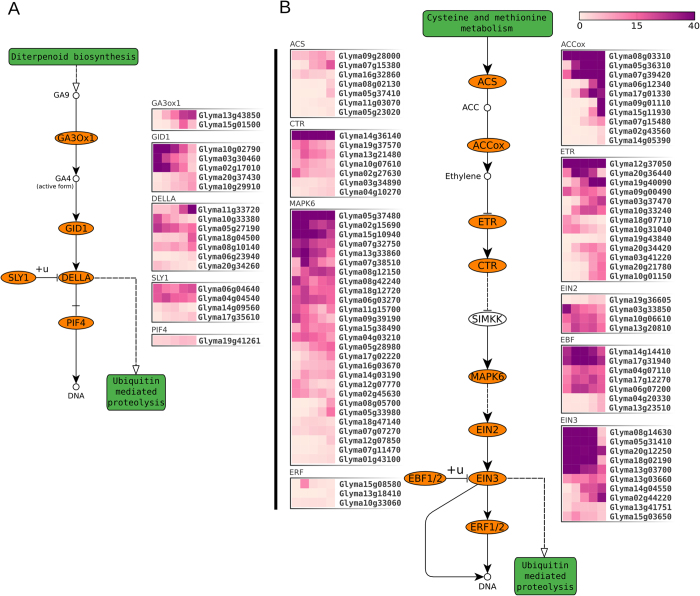
Last biosynthesis steps and core signalling pathways of the germination promoting hormones gibberellin **(A)** and ethylene **(B)**. Schemes were retrieved from KEGG (ko04075). Shapes and arrows follow the KEGG representation standards (http://www.genome.jp/kegg/document/help_pathway.html), except for colour codes. Ellipses represent gene/family. Green rectangles represent connected pathways. Heatmaps represent transcriptional levels (in FPKM) of expressed genes. The 5 columns in each heatmap represent the germination time points (dry; 3-HAI; 6-HAI; 12-HAI; 24-HAI).

GA is a key hormone that promotes seed germination, largely by antagonizing ABA effects^8^. GA binding to the gibberellin-insensitive dwarf1 (GID1) receptor promotes the formation of a DELLA-binding surface^65^,^66^. The GA-GID1-DELLA complex is then recognized by SLEEPY1 (SLY1), which is the F-box component of the SCF^SLY1^ E3 ubiquitin ligase complex. Interaction with SCF^SLY1^ results in DELLA ubiquitination and degradation, releasing GA responses^67^. We found 2 and 5 highly expressed SLY1 and GID1 genes during germination, respectively. Among GID1 genes, 3 are highly transcribed in dry seeds (Fig. 3). Interestingly, these 3 genes belong to the GID1B subfamily, which is induced during after-ripening in the absence of GA biosynthesis^68^. GID1B proteins have higher affinity for GA than GID1A-C^69^ and their overexpression increase GA sensitivity and mimic dormancy-breaking treatments more efficiently than that of GID1A-C^68^. Further, GID1A-C and GID1B group in phylogenetically distinct clades and have different expression patterns during germination in Brassicaceae^70^. Our results are coherent with the functional divergence between GID1A-C and GID1B genes, as the two soybean GID1Cs increase in expression as germination proceeds, as opposed to GID1Bs, which are up to 16-fold down-regulated between 0 and 24-HAI (Fig. 3). Since the high expression of GID1B is not able to compensate the absence of GID1A-C^70^, we hypothesize that the increased expression of GID1C genes is critical to mediate the canonical GA response during soybean seed germination. The high abundance of GID1B transcripts in dry seeds, together with their higher affinity for GA, may be part of a mechanism to perceive very low GA levels promptly upon imbibition, as previously hypothesized^68^. Two SLY1 genes are highly transcribed in all time points (Fig. 3), supporting the capacity of the embryonic axes to sense a wide range of GA levels throughout germination. Finally, we found 7 DELLA genes with distinct expression patterns (Fig. 3). The differential regulation of DELLA and GID1 genes suggest a complex regulatory system, especially because DELLA induces the expression of GID1 and GA biosynthesis genes. Accordingly, we found two highly expressed GA3ox1 genes (Fig. 3), which supports the embryonic axis a major GA biosynthesis site during germination^71^. Since GID1 promotes DELLA proteolysis in the presence of GA, DELLA has been proposed as a central player in GA homeostasis via feedback regulation^72^.

Although not as studied as ABA and GA, ethylene and BR have also been shown to promote seed germination^27^,^73^. As discussed above, important components of the BR pathway are up-regulated during germination (e.g. BRI1). On the other hand, ethylene production starts very early upon imbibition and promotes dormancy release and germination by regulating ABA metabolism and signalling^74^,^75^. The main intermediate of ethylene production is S-adenosyl-methionine (S-AdoMet), which is synthesized from methionine by S-AdoMet synthetase and converted to 1-aminocyclopropane 1-carboxylic acid (ACC) by ACC synthase (ACS). Ethylene synthesis is then catalysed by ACO, which is critical for germination^76^ and presents a high correlation between enzymatic activity and transcript abundance^75^. Further, we found high transcriptional levels of several of these three key ethylene biosynthesis genes throughout germination (Fig. 3). Ethylene can bind to 5 types of transmembrane receptors located at the endoplasmic reticulum: ethylene insensitive 4 (EIN4), ethylene resistant 1 (ETR1), ETR2, ethylene response sensor 1 (ERS1) and ERS2^75^. Genes from all these families, except ERS2, were transcribed in high levels during germination. Further, these results reveal some interesting trends: two ETR1 genes (i.e. *Glyma09g00490, Glyma12g37050*) are highly expressed in all stages, whereas ERS1 and ETR2 genes are generally biased towards the end of germination (Fig. 3) and EIN4 genes display more variable transcriptional profiles (Fig. 3). Ethylene binding to these receptors inactivates CTR1 (constitutive triple response), resulting in the activation of the kinase cascade that controls EIN2 and its nuclear effectors, such as EIN3, EILs and ERFs (ethylene response factors), which will regulate the transcription of ethylene response genes (Fig. 3)^75^. Finally, our results support the feedback regulation in ethylene signalling during soybean germination, as some negative regulators of ethylene response were also highly expressed, such as CTR1 and the F-box EBF1^77^ (Fig. 3).

In the present work we described the sequencing and analysis of the embryonic axis transcriptome during soybean germination. We found a remarkable enrichment of TFs among the genes up-regulated in the onset of germination, especially from the LOB domain family. This suggests that a large set of early TFs play critical roles in triggering the initial transcriptional programs required for germination. We found strong evidence for the transcriptional regulation of several primary metabolic pathways involved in reserve mobilization and ATP production, such as the early activation of respiratory pathways and local degradation of oil reserves to feed the glyoxylate pathway, generally believed to be critical only after germination. We also found evidence for aromatic amino acid biosynthesis at the embryonic axes during germination. Further, several genes involved in the biosynthesis and signalling of the germination-promoting hormones GA, ethylene and BR were up-regulated in distinct stages during germination. Genes involved in ABA degradation and negative regulation were also activated, supporting the notion that the GA:ABA ratio and interplay are critical for germination^9^. Further, we found several cases of strong transcriptional divergence between paralogs in these hormonal regulatory pathways, suggesting that gene duplications play important roles in the regulation of germination. Our results also underscore the importance of post-translational regulatory mechanisms via protein phosphorylation and ubiquitination, which may have important contributions from recent duplications and diversification of F-box genes in the soybean lineage^78^. Taken together, we believe the processes described here shed light on important aspects of the soybean germination and may fuel future biotechnological applications.

## Methods

### Plant material, seed germination and RNA purification

Soybean seeds (cultivar BRS 284) were sown in Petri dishes (14 cm of diameter) containing 2 g of cotton soaked with sterile distilled water (30 mL). Petri dishes were placed into a climate-controlled growth chamber at 28 °C, 12/12 photo-period and 60% relative humidity. In addition to dry seeds, imbibed seeds were harvested at 3, 6, 12 and 24-HAI. Embryonic axes were carefully separated from cotyledons for RNA extraction and weighing. For RNA extraction, collected embryonic axes were immediately placed in RNAlater (Qiagen) on ice until RNA extraction with the RNeasy Plant Mini Kit (Qiagen). Two biological replicates (20 seeds each) of each condition were used. RNA quality was assessed using 1% agarose gel, Picodrop Microliter spectrophotometer and Agilent Bioanalyzer 2100.

### High-Throughput RNA sequencing and analysis

RNA-Seq libraries were prepared using the TruSeq RNA Sample Preparation Kit v2 (Illumina, cat. RS-122-2001) and submitted to paired-end sequencing (2 × 100 bp) using a HiSeq 2000 instrument at LaCTAD (UNICAMP, Campinas, Brazil). Sequencing quality was assessed with *fastqc* (http://www.bioinformatics.babraham.ac.uk/projects/fastqc/). The first 10 bp of each read were trimmed using Trimmomatic-0.30 (option “headcrop = 10”)^79^. The *G. max* reference genome (version 1.1)^3^ was downloaded from Phytozome^80^. Trimmed RNA-Seq reads were mapped to the reference genome using Bowtie 2.1.0^81^ and TopHat 2.0.9^82^. TopHat was executed using the “-b2-very-sensitive” parameters (-D 20 -R 3 -N 0 -L 20 -i S,1,0.50).

Transcriptional levels were estimated with Cufflinks (version 2.1.1; default parameters)^83^ and normalized by fragments per kilobase of transcript per million mapped reads (FPKM). Genes with FPKM equal or greater than 1 were considered “expressed”. Cuffdiff 83 was used to find: a) up-regulated genes between sequential time points (dry vs. 3-HAI; 3-HAI vs. 6-HAI; 6-HAI vs. 12-HAI; 12-HAI vs. 24-HAI) and; (b) genes preferentially expressed at a particular time point. Up-regulated genes were filtered with the Cummerbund^84^ *getSig()* method (*alpha* = *0.1, level* = *‘genes’*); these parameters resulted in differentially expressed genes with at least 2.4 fold-change. Preferentially expressed genes were defined by the *csSpecificity()* function (Cummerbund package), which computes the *Jensen-Shannon distance* (JSD), ranging from 0 (constitutively expressed) to 1 (exclusively expressed). Genes with JSD of 0.7 or greater were defined as “preferentially expressed”. Transcriptional patterns were hierarchically clustered and rendered as heatmaps with Gitools^85^. Additional analyses and parsing steps were performed using in-house perl (https://www.perl.org/) and R (http://www.r-project.org/) scripts. The transcriptome datasets generated in this study were submitted to GEO under the accession number GSE83481.

### Analysis of transcription factors (TFs) and Gene Ontology (GO) enrichment

Soybean TFs and family annotations were retrieved from PlantTFDB^34^ and manually inspected. Enrichment of GO terms was performed using agriGO (FDR < 0.01)^86^. Genes expressed in at least one time point were used as the background set. The final lists of enriched GO terms were compared with SEACOMPARE (agriGO website). Redundancy of the enriched GO terms was removed using REVIGO^87^.

## Additional Information

**How to cite this article**: Bellieny-Rabelo, D. *et al*. Transcriptome analysis uncovers key regulatory and metabolic aspects of soybean embryonic axes during germination. *Sci. Rep.* **6**, 36009; doi: 10.1038/srep36009 (2016).

**Publisher’s note:** Springer Nature remains neutral with regard to jurisdictional claims in published maps and institutional affiliations.

## Supplementary Material

Supplementary Information

## Figures and Tables

**Table 1:** Alignment of RNA-Seq reads on the soybean genome.

Stage	Unmapped reads	Multiple matches	Uniquely mapped	Total mapped reads
Dry	21,888,018	85,630,708	157,064,044	242,694,752
3-HAI	16,427,943	71,875,156	182,185,569	254,060,725
6-HAI	19,798,969	87,809,774	211,697,839	299,507,613
12-HAI	17,874,585	76,704,465	200,731,192	277,435,657
24-HAI	24,183,233	97,381,607	275,170,244	372,551,851

^*^HAI, hours after imbibition.
