# Supplementary material for: Transcriptome analysis uncovers key regulatory and metabolic aspects of soybean embryonic axes during germination
Source: Sci Rep. 2016 Nov 8;6:36009. doi: 10.1038/srep36009 (PMC5099898; doi:10.1038/srep36009)
Supplement: Supplementary Information [file srep36009-s1.pdf]

SUPPLEMENTARY INFORMATION

Transcriptome analysis uncovers key regulatory and metabolic aspects of soybean embryonic axes during germination

Daniel Bellieny-Rabelo, Eduardo Alves Gamosa de Oliveira, Elane da Silva Ribeiro, Evenilton Pessoa Costa, Antônia Elenir Amâncio Oliveira and Thiago Motta Venancio

SUPPLEMENTARY MATERIAL LEGENDS

Figure S1: Global transcriptional patterns of soybean embryonic axes during germination (dry, 3-HAI, 6-HAI, 12-HAI and 24-HAI). Expression patterns were clustered by Hierarchical Clustering. Only expressed genes (FPKM > 1) are represented. FPKM values were converted to z-scores and colour intensities range from -2 to 2. HAI: Hours After Imbibition.

Figure S2: Transcriptional patterns of primary metabolism genes (KEGG database) in soybean embryonic axes during germination (dry, 3-HAI, 6-HAI, 12-HAI and 24-HAI). Only expressed genes (FPKM > 1) are represented. FPKM values were converted to z-scores and colour intensities range from -2 to 2. HAI: Hours After Imbibition.

Table S1: Transcriptional levels (in FPKM) of all soybean protein-coding genes in embryonic axes during germination. Immediately after each FPKM value of each gene, there is a logical (i.e. yes/no) value indicating whether the gene is significantly up-regulated between two subsequent germination phases. Transcription factors are annotated in a separate column. The last three columns contain information of *A. thaliana* orthologs.

Table S2: List of genes showing strong preferential transcription at 24 hours after germination. Information of *A. thaliana* orthologs is also provided.

Table S3: Enrichment of Gene Ontology terms among the differentially expressed genes during germination. These terms were carefully examined along the manuscript, including several single-gene analyses.

Table S4: Transcriptional levels (in FPKM) of the metabolic enzymes represented in figure 2.

SUPPLEMENTARY FIGURES

Figure S1: Global transcriptional patterns of soybean embryonic axes during germination (dry, 3-HAI, 6-HAI, 12-HAI and 24-HAI). Expression patterns were clustered by Hierarchical Clustering. Only expressed genes (FPKM > 1) are represented. FPKM values were converted to z-scores and colour intensities range from -2 to 2. HAI: Hours After Imbibition.

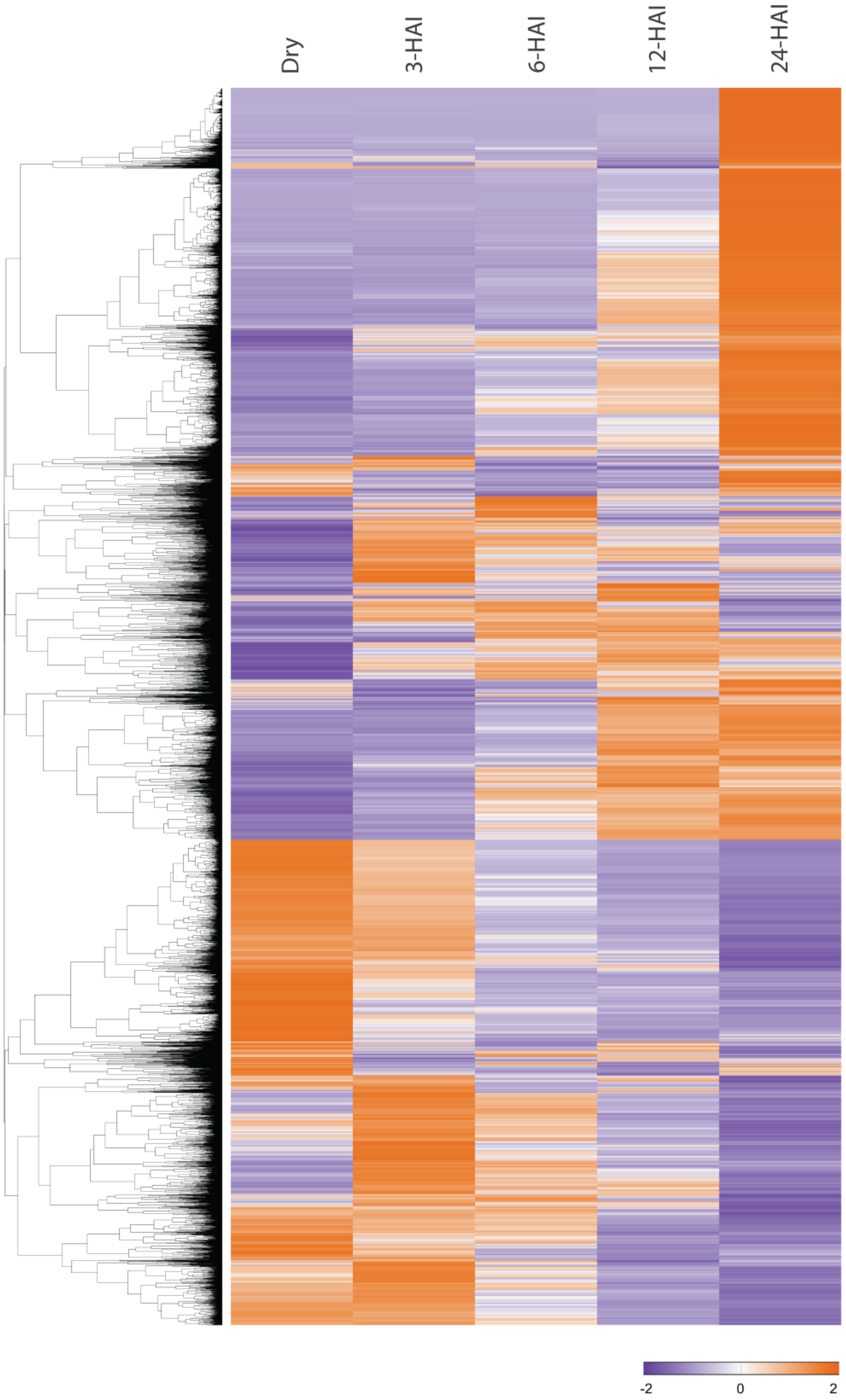

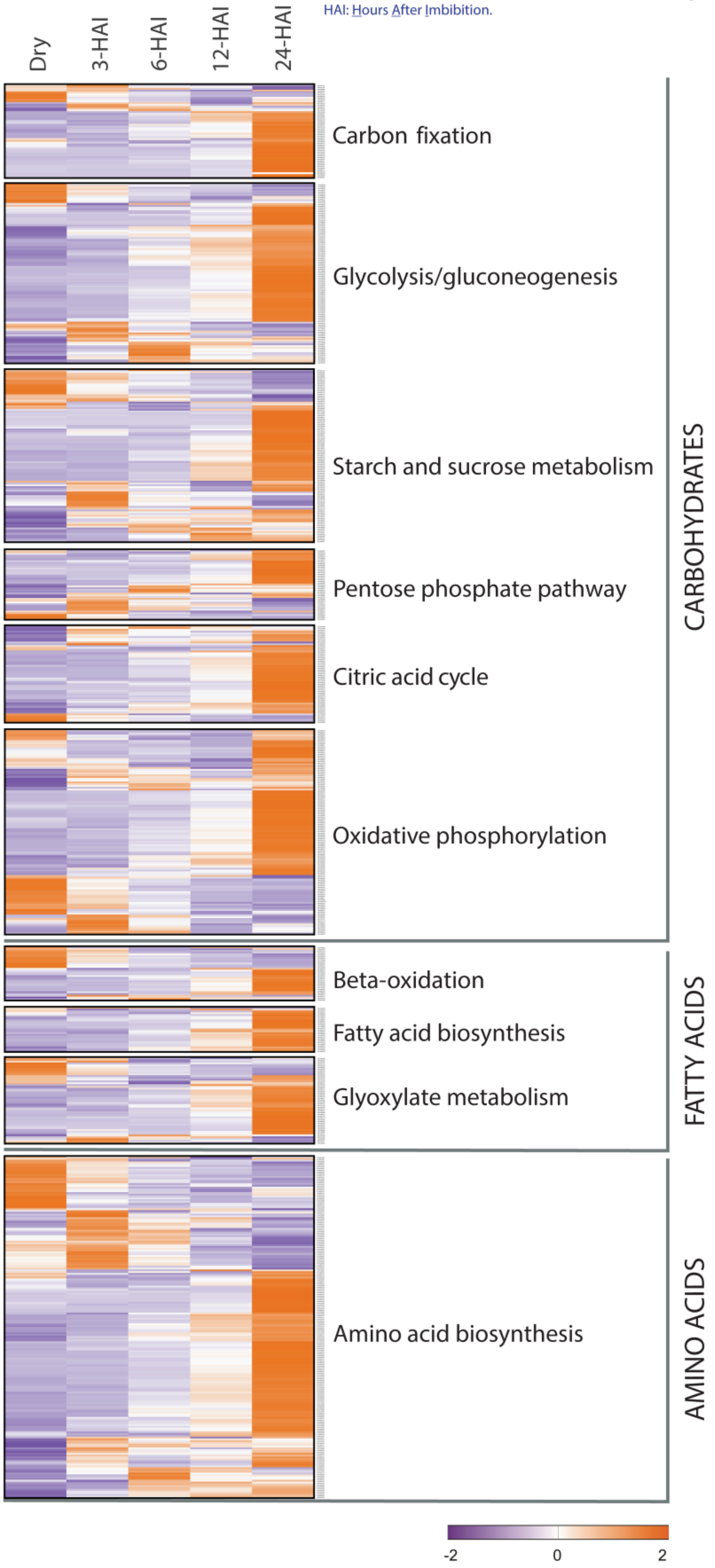

Figure S2: Transcriptional patterns of primary metabolism genes (KEGG database) in soybean embryonic axes during germination (dry, 3-HAI, 6-HAI, 12-HAI and 24-HAI). Only expressed genes (FPKM > 1) are represented. FPKM values were converted to z-scores and colour intensities range from -2 to 2. HAI: Hours After Imbibition.
